# Optimization of fermentation conditions for microbial transglutaminase production by *Streptoverticillium cinnamoneum* KKP 1658 using response surface methodology (RSM)

**DOI:** 10.1007/s12223-024-01223-7

**Published:** 2024-11-23

**Authors:** Vitaliy Kolotylo, Kamil Piwowarek, Alicja Synowiec, Marek Kieliszek

**Affiliations:** Department of Food Biotechnology and Microbiology, Institute of Food Sciences, Warsaw University of Life Sciences—SGGW, Nowoursynowska 159 C, 02-776 Warsaw, Poland

**Keywords:** Transglutaminase, MTG, Streptoverticillium, Response surface methodology

## Abstract

Microbial transglutaminase (MTG) is an enzyme widely used in the food industry because it creates cross-links between proteins, enhancing the texture and stability of food products. Its unique properties make it a valuable tool for modifying the functional characteristics of proteins, significantly impacting the quality and innovation of food products. In this study, response surface methodology was employed to optimize the fermentation conditions for microbial transglutaminase production by the strain *Streptoverticillium cinnamoneum* KKP 1658. The effects of nitrogen dose, cultivation time, and initial pH on the activity of the produced transglutaminase were investigated. The significance of the examined factors was determined as follows: cultivation time > nitrogen dose > pH. The interaction between nitrogen dose and cultivation time was found to be crucial, having the second most significant impact on transglutaminase activity. Optimal conditions were identified as 48 h of cultivation with a 2% nitrogen source dose and an initial medium pH of approximately 6.0. Under these conditions, transglutaminase activity ranged from 4.5 to 5.5 U/mL. The results of this study demonstrated that response surface methodology is a promising approach for optimizing microbial transglutaminase production. Future applications of transglutaminase include the development of modern food products with improved texture and nutritional value, as well as its potential use in regenerative medicine for creating biomaterials and tissue scaffolds. This topic is particularly important and timely as it addresses the growing demand for innovative and sustainable solutions in the food and biomedical industries, contributing to an improved quality of life.

## Introduction

Transglutaminase is an enzyme from the transferase class (EC 2.3.2.13) widely used in the food industry. This enzyme catalyzes three types of protein reactions: (1) acyl transfer reactions, (2) deamidation reactions, and (3) cross-linking reactions between glycine and lysine residues (Kieliszek and Misiewicz 2014; Kolotylo et al. 2023). Transglutaminase has applications in various industries, including packaging (bioplastics) (Mirpoor et al. 2024), dairy (Hidas et al. 2023; Marhons et al. 2023; Romeih et al. 2024), fish (Tokay et al. 2021), meat (Feng et al. 2024b, a), and bakery (Schlangen et al. 2023; da Ramos et al. 2023).

Initially, commercial transglutaminase was derived from animal sources, but it has now been completely replaced by microbial transglutaminase (MTG) (Redd et al. 2024). Microbial transglutaminase has entirely replaced the animal-derived enzyme because it is cheaper and more efficient to produce, exhibits higher stability and activity under various conditions, is safer for consumers, and is more widely accepted ethically and religiously. Furthermore, creating the enzyme through microorganisms allows for better quality control, ensuring a uniform and pure final product (Motoki and Seguro 1998; Yokoyama et al. 2004). Currently, transglutaminase is primarily obtained from the strain *Streptomyces mobaraensis*. It is also produced by other microorganisms such as *Streptoverticillium lydicus*, *Streptoverticillium ladanum*, *Bacillus subtilis*, and *Bacillus sphaericus* (Ceresino et al. 2018; Kolotylo et al. 2023). Enzymes are crucial for the processing industry owing to their specificity. Such metabolites can minimize by-products in the agro-food industry, act quickly, and require moderate reaction conditions. The industrial enzymes market has grown consistently in both production and revenue (Tarafdar et al. 2021). Enzyme sales in the USA amounted to $1.3 billion in 2002 and $5.1 billion in 2009, with food enzymes constituting 31% and feed enzymes 6% of the total market (Sarrouh et al. 2012). As of 2019, the enzyme market was projected to reach a value of $10.6 billion in 2020, with an annual growth rate of 7.1%, reaching approximately $14.9 billion by 2027. This growth is attributed to the importance of enzymes in the food, biofuel, agricultural, detergent, textile, and cosmetic industries. The food industry holds the largest share of the enzyme market, where enzymes are used for producing food additives and dietary supplements, in beverage industries, vegetable and fruit processing, cheese, protein, grains, oils, fats, and dairy products, as well as in brewing, baking and agriculture (Wu et al. 2020). Thus, optimizing enzyme production processes is crucial for ensuring sustainable development.

Response surface methodology (RSM) is an effective technique for optimizing biotechnological processes, relying on a strategy for finding optimal conditions in multidimensional systems. RSM is preferred over classical optimization methods because it allows simultaneous examination of the effects of multiple variables and their interactions on the outcome, which reduces the number of necessary experiments, saves time and resources, and increases accuracy through its statistical approach. Compared to other methods, RSM offers a more comprehensive and effective tool for optimizing multivariable processes, allowing for better modelling, control, and visualization of data, which is crucial in advanced scientific research (Bezerra et al. 2008). This method has been successfully applied to optimize the composition of the medium for xanthan production and conditions for enzymatic hydrolysis since the 1990s (Ma and Ooraikul 1986; Roseiro et al. 1992). In 2010, the method was used to optimize pullulan production (Jiang 2010); in 2023, to maximize emulsion stability (Liu et al. 2023); and in 2024, studies were published on optimizing the extraction process of sugars and phenolic compounds (De la Lama-Calvente et al. 2024). Selected studies using this optimization method over the years demonstrate its timelessness and continued relevance, which is why it was utilized in the present study.

This study employs response surface methodology to determine the combined effect of three crucial cultivation parameters—nitrogen dose, cultivation time, and initial medium pH—on transglutaminase production by *Streptoverticillium cinnamoneum* KKP 1658.

## Materials and methods

### Microorganism

The study was conducted using the actinomycete strain *Streptoverticillium cinnamoneum* KKP 1658, stored in the Pure Culture Collection of the Department of Biotechnology and Food Microbiology at the Warsaw University of Life Sciences. The actinomycetes were maintained at − 80 °C in tryptic soy broth with glycerol (Chempur, Poland). It is worth noting that the *S. cinnamoneum* KKP 1658 strain was purchased from the Institute of Agricultural and Food Biotechnology State Research Institute (IAFB) Collection of Industrial Microorganisms (Warsaw, Poland). This collection contains a very large number of microorganisms that are identified, shared, and stored long-term by the global standards of the World Federation for Culture Collection (WFCC), the guidelines of the European consortium CABRI (Common Access to Biological Resources and Information), and the recommendations of the OECD (OECD Best Practice Guidelines for Biological Resource Centres) regarding best practices in the storage of biological materials.

### Preparation *of medium*

The inoculation medium TSB (tryptic soy broth) contained (g/L): casein peptone (BioMaxima, Poland) 17, soy peptone (BioMaxima, Poland) 3, NaCl (P.P.H. Stanlab, Poland) 5, K_2_HPO_4_ (POCH S.A., Poland) 2.5, glucose (Chempur, Poland) 2.5. The inoculation medium was sterilized at 121 °C for 15 min (Systec D-45 autoclave, De Ville, Poland) after adjusting the pH to 7.0 (pH meter Elmetron CP-505, Poland).

To optimize the production process of microbial transglutaminase, the effects of three different nitrogen source doses (1%, 2%, and 4% in the form of a combination of amino-bak (BTL, Poland) and corn steep liquor (Massive baits, Poland) in a 1:1 ratio) and pH levels of 5.5, 6.0, and 6.5 were investigated (Kolotylo et al. 2024). The composition of the research medium with 2% nitrogen source content (g/L) was as follows: soluble starch (Chempur, Poland) 20, amino-bak (BTL, Poland) 10, corn steep liquor (Massive baits, Poland) 10, yeast extract (BioMaxima, Poland) 2, KH_2_PO_4_ (POCH S.A., Poland) 2, Na_2_HPO_4_ (POCH S.A., Poland) 2, MgSO_4_ 7H_2_O (Biomus, Poland) 1. The research media were sterilized at 121 °C for 15 min (autoclave) after adjusting the pH to 5.5, 6.0, or 6.5.

### Cultivation methods

The starter culture was prepared by inoculating a 500-mL flask containing 100 mL of TSB medium with cells of the test strain (6–8 × 10^7^ CFU/mL) and incubating at 28 °C for 72 h on a shaker (Eppendorf Innova 44 Incubator Shaker, Germany) at 180 rpm. Then, 10 mL of the starter culture was transferred to 90 mL of fresh TSB medium to obtain the inoculation culture, which was incubated for 24 h at 28 °C and 180 rpm. For the production of microbial transglutaminase, 10 mL of the inoculation medium was transferred to 90 mL of research media. Cultures were incubated at 28 °C and 180 rpm for three different time periods: 24, 48, and 72 h.

### Biomass yield determination

The biomass yield of *Streptoverticillium cinnamoneum* KKP 1658 was determined after centrifuging (Eppendorf 5810 Centrifuge, Germany) (4500 rpm, 10 min) 30 mL of the culture broth in dry, pre-weighed Falcon tubes. The supernatant was decanted for transglutaminase activity determination, and the sediment was dried (SML Zalmed Dryer, Poland) (80 °C) to a constant mass. Biomass yield after centrifugation and drying in triplicate was calculated per 1 L of medium and expressed in grams of dry substance (g of dry substance per L).

### Transglutaminase activity determination

Transglutaminase activity was determined using commercial test kits Microbial Transglutaminase Assay Kit Art. No. Z009 (Zedira GmbH, Darmstadt, Germany). The assay uses Z-Gln-Gly (N2-[(phenylmethoxy)carbonyl]-L-glutaminyl-glycine, C15H19N3O6) (Zedira GmbH, Germany) as the amine acceptor and hydroxylamine (Zedira GmbH, Germany) as the amine donor. In the presence of MTG, hydroxylamine is incorporated into the Z-Gln-Gly substrate, forming Z-glutamyl-hydroxaminyl-glycine, which forms a colored complex with iron (III) detectable spectrophotometrically (BIO-RAD SmartSpec 3000 Spectrophotometer, Poland) at a wavelength of 525 nm (Kolotylo et al. 2024).

### Analytical methods

The statistical optimization of parameters affecting microbial transglutaminase production was conducted using a Box–Behnken design, which employs three levels of three different factors. Statistical analysis, experimental design, and model building were performed using Statistica software version 13.1.

## Results and discussion

Microbial transglutaminase (MTG) is highly esteemed in the food sector for its ability to modify protein structures without altering the product’s taste, color, or nutritional value. By cross-linking proteins, this enzyme enhances water retention, texture, and firmness in a range of products, including meat, fish, dairy, and baked goods (Kolotylo et al. 2023). One of the earliest applications of MTG was in the production of surimi, where it improves texture and elasticity by cross-linking myosin in fish muscle, giving the product its essential gel-like (Yokoyama et al. 2004; Romeih and Walker 2017). In the meat industry, MTG facilitates smaller pieces of meat to be bound together into high-quality products like sausages and restructured steaks, enhancing cohesiveness and texture without the need for added salt (Duarte et al. 2019a). In baking, the enzyme increases dough elasticity, boosts the volume of baked goods, and extends shelf life, which is particularly beneficial for gluten-free products (Šmídová and Rysová 2022). In dairy processing, MTG improves the stability and texture of yogurts and cheeses by enhancing water-binding capacity and reducing whey separation (Kieliszek and Błażejak 2017). Beyond the food industry, MTG finds use in various other sectors.

In textiles and leather manufacturing, the enzyme enhances the durability and elasticity of protein-based materials like wool by cross-linking proteins, improving fiber strength and resistance to shrinkage (Yokoyama et al. 2004; Yokoyama 2024). In biotechnology, MTG is employed in developing new biomaterials, such as wound dressings and scaffolds for tissue engineering, thanks to its ability to form durable, water-insoluble protein structures—crucial for applications requiring high mechanical stability (Xia et al. 2022; You et al. 2024).

In the context of increasing demand for efficient and economical enzyme production methods, our work focuses on identifying and optimizing key factors influencing the yield of transglutaminase production by *Streptoverticillium cinnamoneum* KKP 1658. It is worth emphasizing that the *Streptoverticillium cinnamoneum* strain (syn. *Streptomyces cinnamoneus*, *Streptomyces sapporonensis*) (Hatano et al. 2003) exhibits an aerobic metabolism typical of actinomycetes. It shows the ability to assimilate various carbon substrates, which makes it a versatile microorganism in various environments. Therefore, it can be found in soil (Gopalakrishnan et al. 2020), where it plays an essential role in decomposing organic matter and recycling nutrients. It mainly prefers soil rich in organic carbon compounds. The *S. cinnamoneum* genome is rich in genes responsible for synthesizing secondary metabolites, which is reflected in the ability of this organism to produce a wide range of bioactive compounds. A high percentage of guanine and cytosine in DNA is characteristic of actinomycetes, which correlates with their adaptation to difficult environmental conditions (Gopalakrishnan et al. 2020). One of the key features of *S. cinnamoneum* is its ability to biosynthesize antibiotics (Paradkar et al. 1998), especially from the aminoglycoside group, including cinnamycin. Cinnamycin, as a secondary product, not only plays a vital role in the microorganism’s defense strategies, but also has potential applications in antibacterial therapy (Ramírez-Rendón et al. 2023). The production of transglutaminase by this strain is also noteworthy, as this enzyme has applications in biotechnology (Cabrera-Barjas et al. 2022), including the food and pharmaceutical industries, where it is used to modify proteins and improve the textural properties of foods (Velazquez-Dominguez et al. 2023). In summary, *Streptoverticillium cinnamoneum* is a microorganism of great ecological and biotechnological value, whose unique metabolic properties and ability to produce bioactive compounds make it the subject of intensive research in the field of biotechnology.

The use of response surface methodology (RSM) allows for the systematic examination of the impact of various process parameters and their interactions, enabling the determination of optimal cultivation conditions. The following results and discussion elucidate the production process and suggest improvements that can enhance yield and reduce the production costs of transglutaminase. To find the optimal cultivation conditions for *Streptoverticillium cinnamoneum* KKP 1658 in batch cultures, it is essential to identify the key factors influencing microbial transglutaminase production. Our previous experiments (Kolotylo et al. 2024) determined the main factors influencing transglutaminase production by the strain *S. cinnamoneum* KKP 1658. Table 1 presents the selected ranges of nitrogen source doses (*A*), cultivation time (*B*), and initial pH (*C*) (coded as − 1, 0, and 1, where − 1 denotes the minimum value of the given range and 1 the maximum value of the range) used in this study.

**Table 1:** Values of coded levels used for the experimental design

Factors (independent variables)	Symbols	Actual levels of coded factors		
		** − 1**	**0**	**1**
Dose of nitrogen source (%)	A	1	2	4
Time (h)	B	24	48	72
Initial pH	C	5.5	6.0	6.5

To optimize the conditions for transglutaminase production by *Streptoverticillium cinnamoneum* KKP 1658, an experiment was designed following the principles of the three-level fractional factorial designs according to Box–Behnken for three factors. In total, 15 experiments were conducted, consisting of 12 different combinations of the three factors and three repetitions at the central point. The experimental data were used to generate the coefficients of the quadratic polynomial model and response surface plots were constructed for each dependent variable to visualize the response surface field. The experimental data for the Box–Behnken statistical plan are presented in Table 2. The regression coefficients were calculated, and the fitted equations to predict transglutaminase activity (*X*) are given below:

1 $$X=-294.69+112.76\times A-4.88\times A2+96.62\times C-8.08\times C2-34.45\times A\times C+2.69\times A\times C2+0.71\times A2\times C-0.02\times B\times A+0.002\times B\times A2+0.03\times B\times C+1.74$$

where *A* is the nitrogen source dose, *B* is the cultivation time, and *C* is the initial pH of the culture.

**Table 2:** Box–Behnken design with actual and coded levels of variables and actual values of responses

Run numbers	Nitrogen source (%)	pH	Time (h)	Biomass yield (g _dry mass_/L)	Transglutaminase activity (U/mL)
1	1	5.5	48	12.02 ± 0.36	2.812 ± 0.50
2	4	5.5	48	17.25 ± 0.52	1.354 ± 0.24
3	1	6.5	48	14.93 ± 0.45	2.332 ± 0.42
4	4	6.5	48	22.75 ± 0.68	5.085 ± 0.91
5	1	6.0	24	11.55 ± 0.35	1.739 ± 0.31
6	4	6.0	24	14.26 ± 0.43	0.819 ± 0.15
7	1	6.0	72	11.06 ± 0.33	3.114 ± 0.56
8	4	6.0	72	18.92 ± 0.57	1.288 ± 0.23
9	2	5.5	24	15.62 ± 0.47	2.476 ± 0.44
10	2	6.5	24	14.36 ± 0.43	1.350 ± 0.24
11	2	5.5	72	16.03 ± 0.48	2.687 ± 0.48
12	2	6.5	72	16.58 ± 0.50	2.829 ± 0.50
13	2	6.0	48	18.71 ± 0.56	4.595 ± 0.82
14	2	6.0	48	17.22 ± 0.52	4.348 ± 0.78
15	2	6.0	48	18.42 ± 0.55	4.561 ± 0.81

**Fig. 1:**
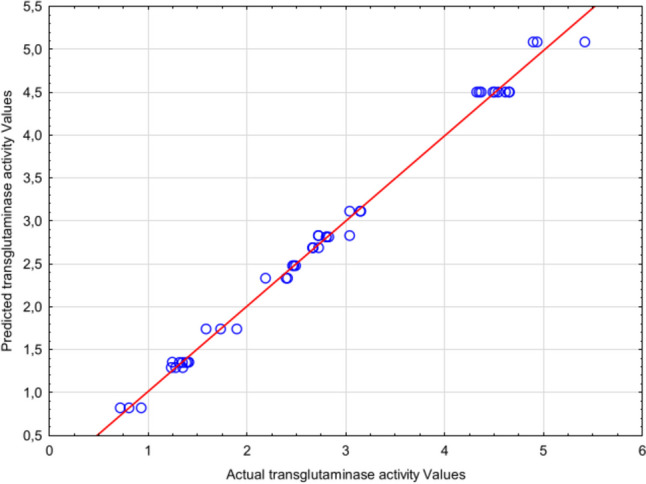
Predicted vs actual values for transglutaminase activity

The predicted values calculated from the above equation showed excellent agreement with the experimental data, as shown in Fig. 1, indicating the suitability of this quadratic model for the present experimental setup.

The fit of the experimental model to the obtained transglutaminase activity results was expressed by the coefficient of determination *R*^2^. In this case, the *R*^2^ value of 99.4% indicates that the response model can explain over 99% of all variance. According to Jiang (2010), any regression model with a coefficient of determination exceeding 0.9 (90%) has a very high correlation. After adjustment, the *R*^2^_Adj_ value decreased to 99.1%, but remained sufficiently high to indicate a good fit of the model to the obtained data. According to Liu et al. (2023), for a model to be considered appropriate, the difference between the adjusted *R*^2^Adj and the predicted *R*^2^ values should be within the range of 0 to 0.200. The difference between these values in the present study is 0.0025, indicating the model’s high accuracy in predicting response values.

A significance level of 0.05 (*p*-value) was used to determine the significance of the model factors, where values above this threshold are considered non-significant, and values below indicate significance (Table 3). Using the *F*-value, we can determine the degree of influence of the studied factors on the activity of the produced transglutaminase. Very low probability values (*p*-value < 0.0001) suggest that high *F*-values are not due to random noise, which is an inherent part of laboratory experiments (Liu et al. 2023).

**Table 3:** Analysis of variance for response surface quadratic model obtained from experimental results

Source	Sum of squares	df	Mean squares	*F*-value	*p*-value
A	0.00370	1	0.00370	0.233	0.632932
B	3.78148	1	3.78148	237.585	< 0.0001
C	7.02731	1	7.02731	441.515	< 0.0001
A^2^	11.3970	1	11.3970	716.060	< 0.0001
B^2^	23.4632	1	23.4632	1474.162	< 0.0001
C^2^	1.19379	1	1.19379	75.004	< 0.0001
AB	0.61438	1	0.61438	38.600	< 0.0001
AC	13.3009	1	13.3009	835.675	< 0.0001
BC	1.20560	1	1.20560	75.746	< 0.0001
AC^2^	6.11818	1	6.11818	384.395	< 0.0001
A^2^C	2.84513	1	2.84513	178.755	< 0.0001
A^2^B	0.07356	1	0.07356	4.622	0.0392
Pure error	0.50932	32	0.01592	**-**	**-**
Core total	78.0273	44	**-**	**-**	**-**
*R*^2^	0.9935	-	**-**	**-**	**-**
*R*^2^_Adj_	0.9910	-	**-**	**-**	**-**

**Fig. 2:**
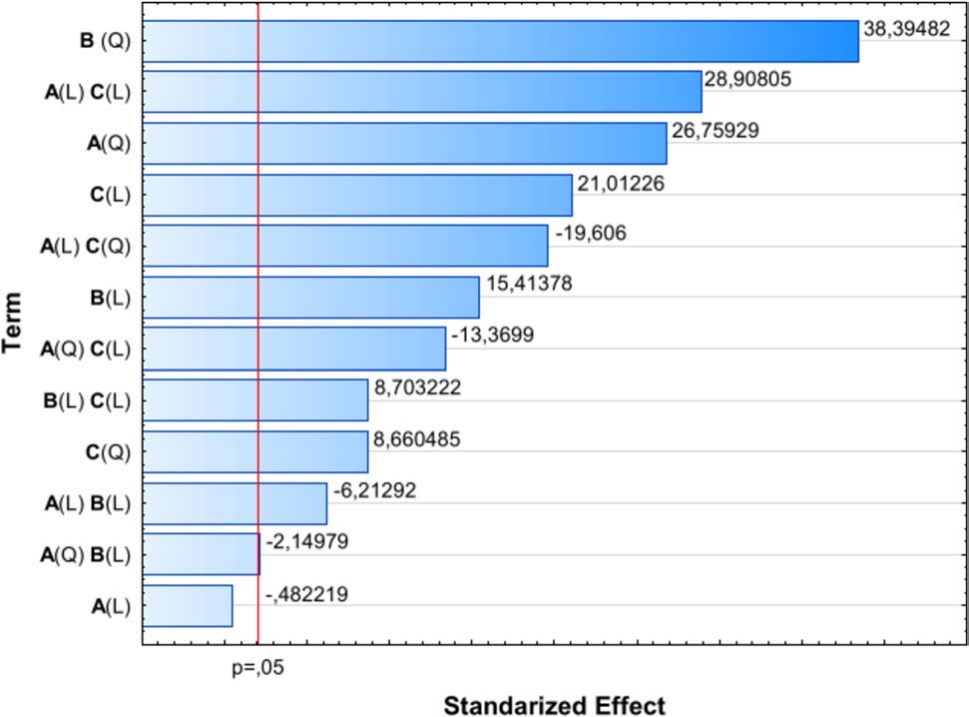
Pareto chart of standardized effect (response is transglutaminase activity (U/mL)). *A* represents nitrogen source dosage; *B* represents cultivation time; *C* represents initial pH. *L* denotes linear effects, while *Q* denotes quadratic effects

To facilitate the visualization of the influence of individual components in the determined Eq. (1), a Pareto chart was created, displaying the absolute values of the standardized Student’s *t*-test and including a line corresponding to a significance level of 0.05 (Fig. 2).

**Fig. 3:**
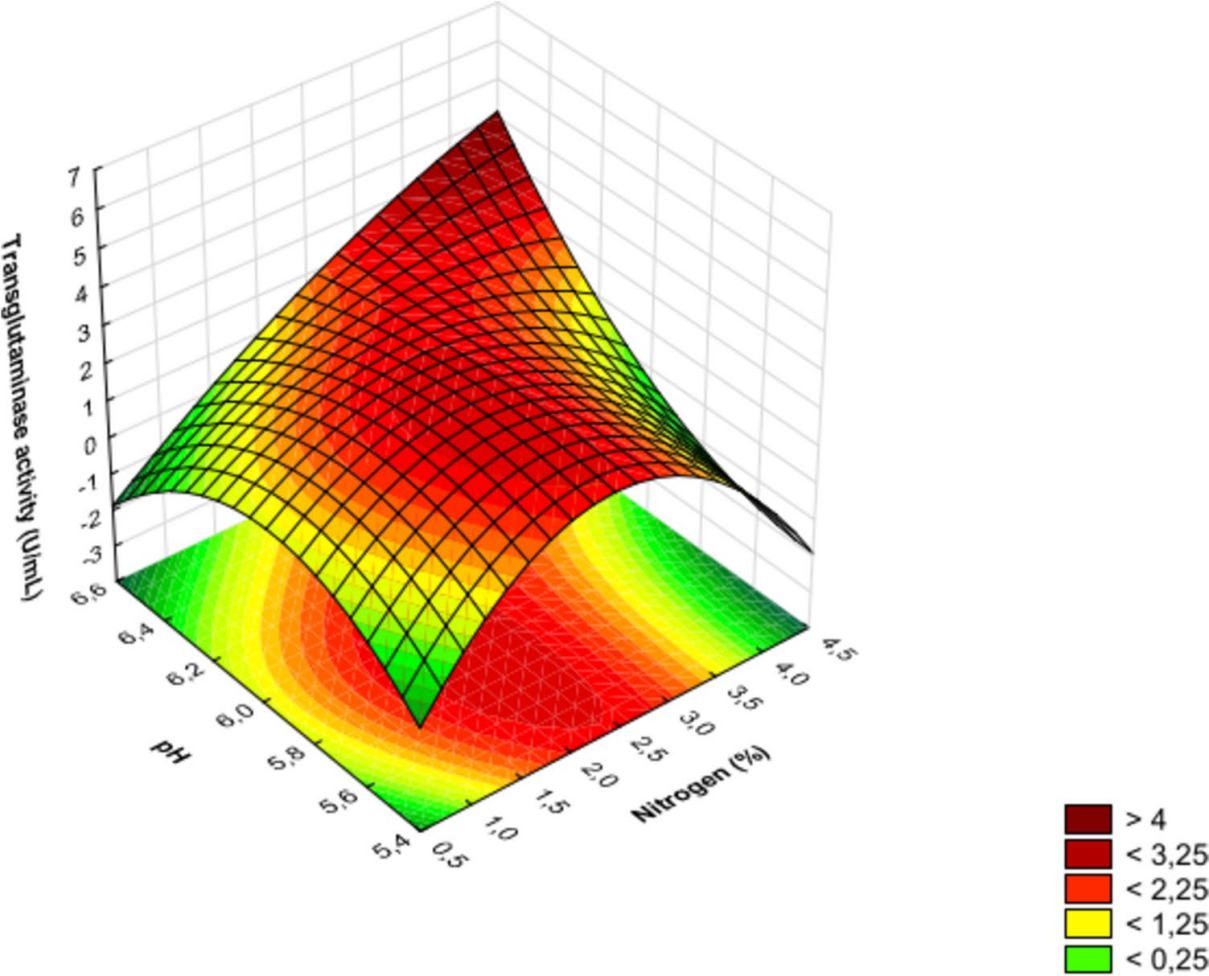
Response surface for MTG production by *Streptoverticillium cinnamoneum* KKP 1658 after 24 h of culture. The interaction between the dose of nitrogen source and initial pH

**Fig. 4:**
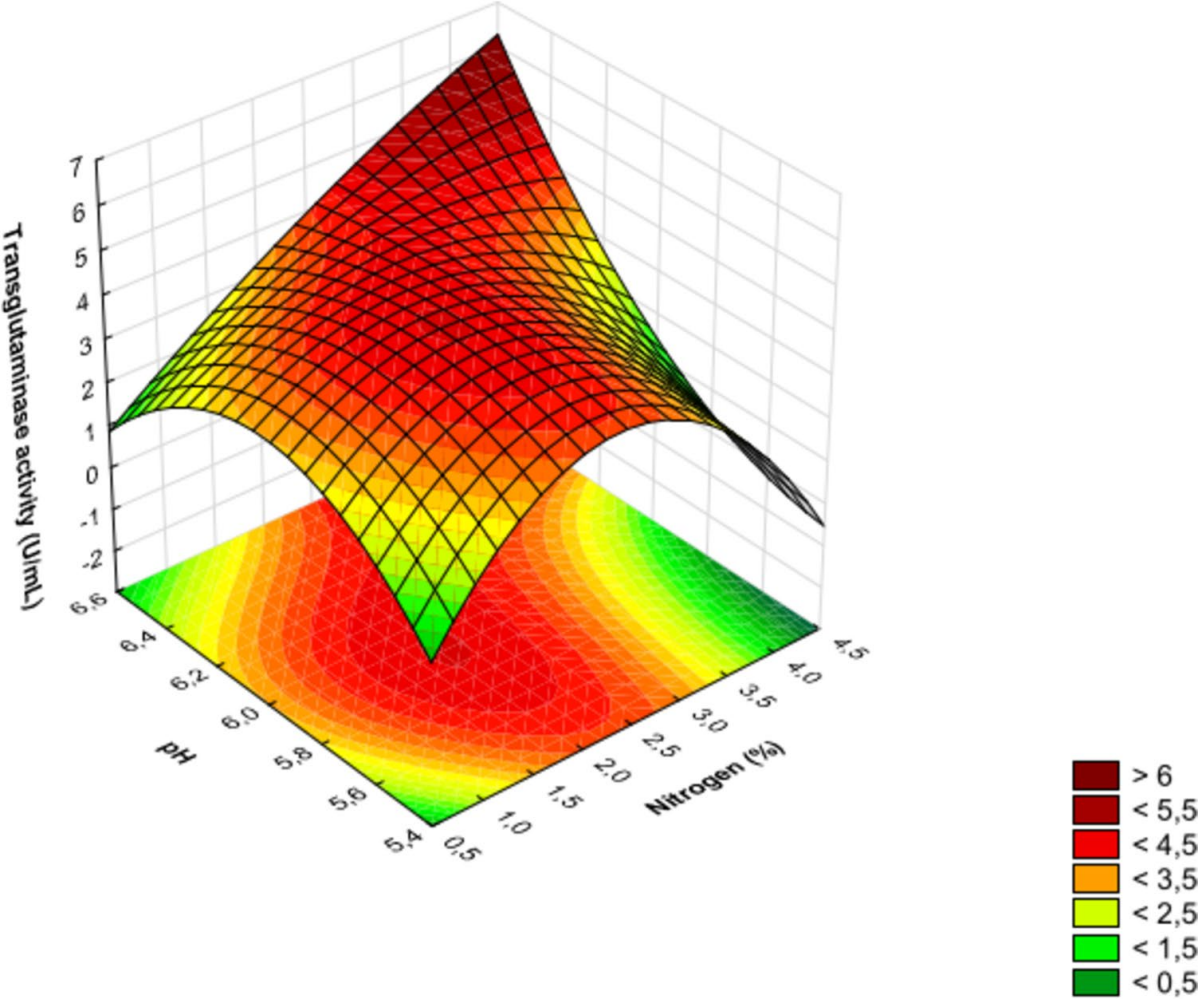
Response surface for MTG production by *Streptoverticillium cinnamoneum* KKP 1658 after 48 h of culture. The interaction between the dose of nitrogen source and initial pH

**Fig. 5:**
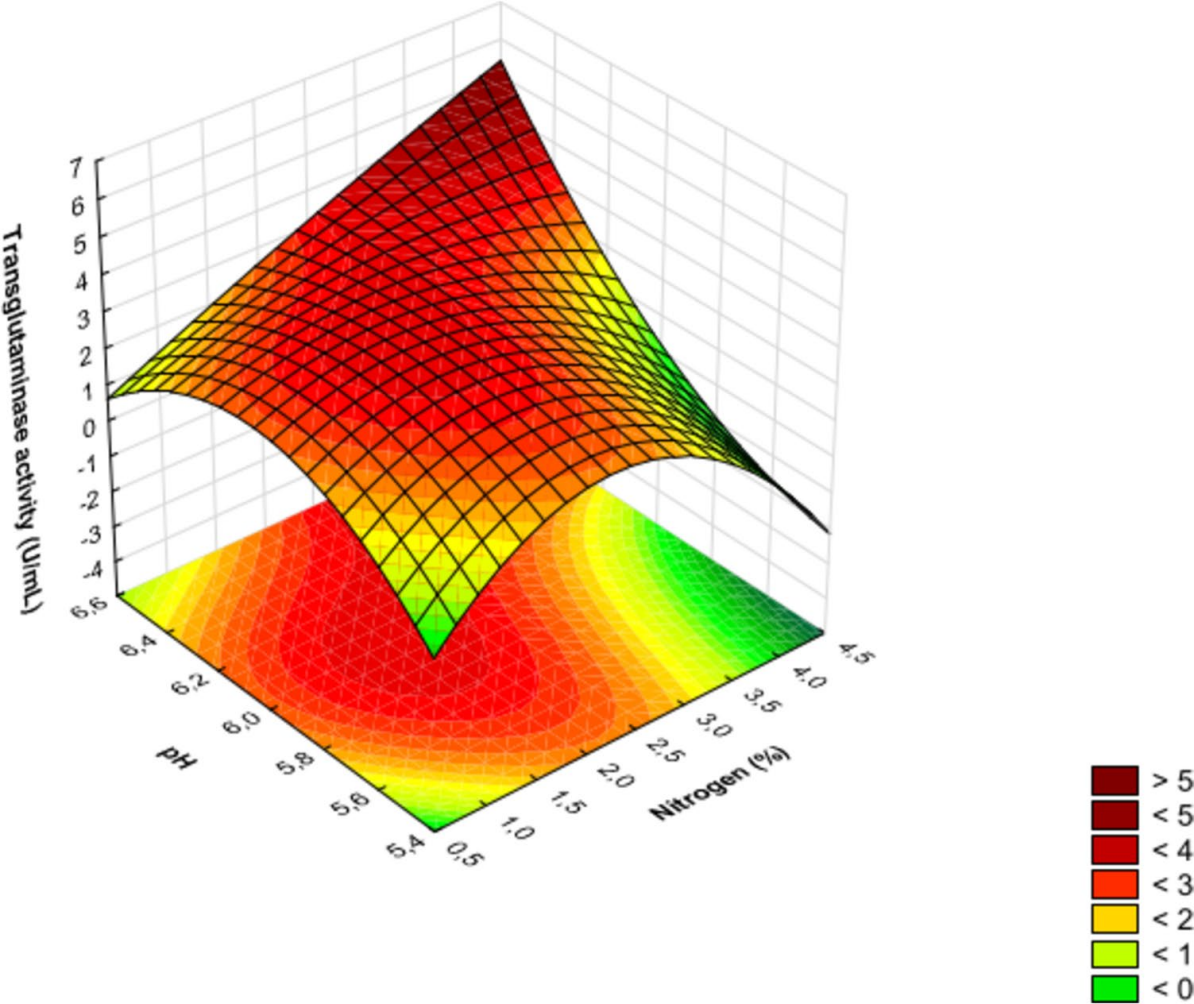
Response surface for MTG production by *Streptoverticillium cinnamoneum* KKP 1658 after 72 h of culture. The interaction between the dose of nitrogen source and initial pH

In the experiment discussed, the linear effect of factor *A* (nitrogen source dosage) was the only one found to be insignificant, while the quadratic effect of this factor ranked third in terms of the absolute value of the standardized Student’s *t*-test. For each investigated factor (Table 1) affecting the production of transglutaminase by *S. cinnamoneum* KKP 1658, linear, nonlinear (quadratic), and interaction effects are presented in Table 3 and Fig. 2. The significant order in which the examined factors linearly influence the transglutaminase activity is as follows: initial pH (*C*) > cultivation time (*B*) > nitrogen source dosage (*A*). The quadratic effects are ordered as follows: cultivation time (*B*) > nitrogen source dosage (*A*) > pH (*C*). The results show that the most significant influence on MTG production is the quadratic effect of factor *B* (cultivation time), indicating that the highest yield is achieved around 48 h of cultivation. It is also important to note the impact of the linear interaction between factors *AC* (nitrogen source dosage and cultivation time), which, according to statistical analysis, had the second most significant effect on the obtained MTG activity. Figures 3, 4, and 5 present three-dimensional surface response plots of the predictive quadratic model.

Three-dimensional response surface plots were utilized to determine the optimal conditions for transglutaminase production. Cultivation of *S. cinnamoneum* KKP 1658 for 24 and 72 h led to MTG production with activity not exceeding 4 U/mL (Figs. 3 and 5). Additionally, it was observed that transglutaminase activity values > 4 U/mL were obtained after experimental cultivation (48 h), which is confirmed by actual results (Table 2) and those predicted by the model (Fig. 4). The highest MTG activities (> 6 U/mL) after 48 h of cultivation (Fig. 4) were found in areas corresponding to an initial pH range of 6.4–6.6 and high nitrogen source dosages > 4%.

A crucial question arises: are the parameters that achieved the maximum transglutaminase activity optimal? The answer to this question is that the parameters (nitrogen source dosage > 4% and pH range 6.4–6.6) that achieved the maximum MTG activity were not optimal. Figure 4 shows that using half the nitrogen dosage (2%) and a pH range of 5.8–6.0 can achieve transglutaminase activity levels of 4.5–5.5 U/mL, which is still very promising. Optimization can be defined as studies based on technical and economic feasibility, which in turn is achievable by maximizing yield while minimizing raw material costs (Lima et al. 2023). According to Akbari et al. (2021), maintaining economic benefits in transglutaminase production is associated with reducing the use of expensive substrates (aminobac, peptone, yeast extract). Studies by Kolotylo et al. (2024) confirmed that replacing half of the aminobac dose with waste corn steep liquor not only reduces the costs associated with this expensive microbiological substrate by 50% but also increases MTG production by the *S. cinnamoneum* KKP 1658 strain. The authors report that for media with only aminobac or only corn steep liquor, transglutaminase activity > 2 U/mL was not detected within 96 h of cultivation. Combining these two components in a 1:1 ratio yielded MTG activity of 6.59 U/mL, which according to Kolotylo et al. (2023) is one of the highest values in the available literature for non-genetically modified strains.

Establishing the optimal pH is critical for the conformational stability of microbial transglutaminase, which directly impacts its catalytic activity. Enzymes such as MTG require specific pH conditions to maintain the proper folding of their active sites, which is crucial for efficient substrate binding and catalysis. Deviations from the optimal pH can destabilize the enzyme’s tertiary structure, resulting in a significant loss of activity. Research indicates that an optimal pH of around 6.0 for MTG supports maximum conformational stability, which preserves its functional integrity and enhances enzymatic cross-linking efficiency (Shi et al. 2011; Vasić et al. 2023). This alignment between structural stability and optimal catalytic performance underscores the importance of carefully controlling pH during fermentation.

Additionally, the fermentation temperature of 28 °C employed in this study appears to foster robust cell growth and efficient enzyme production. This temperature is close to the physiological optimum for many *Streptoverticillium* species, promoting bacterial proliferation and the active transcription and translation of the MTG gene. Such conditions are likely to optimize metabolic activity, thereby enhancing the yield of MTG (Duarte et al. 2019b). These findings suggest that the chosen temperature provides a dual benefit by maximizing both microbial biomass and enzymatic output, critical factors in scaling up the production of industrially relevant enzymes.

*Streptoverticillium cinnamoneum* is widely regarded as a strong producer of microbial transglutaminase (MTG) in the literature, mainly due to its frequent use in the development of recombinant strains for industrial enzyme production. This strain’s ability to generate high yields of MTG makes it a valuable asset in biotechnological applications, especially in the food industry (Kolotylo et al. 2023, 2024). A study by Noda et al. (2012) exemplifies this by using *Streptomyces lividans* as a recombinant host for the production of MTG from *S. cinnamoneum*. The researchers found that *Streptomyces lividans* successfully produced significant amounts of MTG when cultured on various biomass-derived carbon sources, achieving the highest yield of 530 mg/L with xylose as the carbon source. This highlights the potential of *S. cinnamoneum* in MTG production, showcasing its adaptability to various substrates and its promising application in optimizing industrial enzyme production processes.

The study by Tokai et al. (2020) investigated the substrate recognition mechanisms of microbial transglutaminases, specifically comparing enzymes from *Streptomyces mobaraensis* (SMTG) and *Streptomyces cinnamoneum* (SCTG). The primary objective was to understand how these enzymes interact with natural peptides, particularly in food processing contexts. The researchers found that although SCTG is structurally similar to SMTG, it exhibited different activity profiles, with SCTG displaying optimal activity at pH 8.0 and a lower substrate affinity compared to SMTG. In the context of our research, these findings are particularly relevant when discussing the pH-dependent conformational stability of MTG. The observed optimal pH of 8.0 for SCTG in Tokai’s study, although slightly higher than the optimal pH identified in our research, still supports the concept that pH plays a critical role in maintaining the enzyme’s active site conformation, which is essential for catalytic efficiency. Our results, which emphasize the importance of optimal pH for maximizing MTG activity, align with these findings.

Additionally, the temperature condition of 28 °C used in our experiments is similarly advantageous for microbial growth and enzyme production. This is consistent with Tokai et al.’s results, where SCTG expression and enzymatic activity were most stable at moderate temperatures around 30 °C. This analysis underscores the importance of carefully controlling pH and temperature for both microbial viability and the catalytic function of transglutaminase, highlighting the potential for optimizing enzyme production on an industrial scale.

The study by Junqua et al. (1997) aimed to optimize microbial transglutaminase production in *Streptoverticillium cinnamoneum* CBS 683.68 and investigate the relationship between microbial growth and MTG activity. They employed a series of experimental designs (factorial, Box-Behnken, and composite designs) to identify the effects of various factors—such as casein, glycerol, and yeast extract-on MTG production and biomass growth. The research showed that casein and glycerol were the most significant factors, with the combination of these two components leading to a threefold increase in MTG activity, reaching a maximum of 0.331 U/mL. Additionally, transglutaminase production was primarily observed during the stationary growth phase, suggesting that enzyme production could be induced under specific nutrient conditions. The study by Junqua et al. (1997) highlights the critical importance of selecting the appropriate carbon and nitrogen sources to optimize microbial transglutaminase production processes. Their findings demonstrate that casein and glycerol were the most influential factors for MTG activity and microbial growth.

Furthermore, the observation that MTG production was strongly associated with the stationary growth phase provides important insights into the timing of enzyme activity. This aligns with our results, where the highest MTG activity was observed after 48 h of cultivation, a time point corresponding to the stationary phase of cell growth in our system. These complementary findings emphasize the importance of optimizing nutrient conditions and timing in bioprocesses to maximize enzyme yields.

In recent years, waste or renewable raw materials have become a key element of industrial production processes and sustainable development (Beltrán-Ramírez et al. 2019). This approach allows the efficient valorization of agricultural waste into high-value biotechnological products, which is extremely important for the environment, and supports entrepreneurs by somewhat reducing production costs (Lima et al. 2023). Other by-products from the agri-food industry that can be used in microbial media include sugarcane molasses, wheat and oat bran, soybean meal, potato juice, potato pulp, and fruit flour (Preichardt et al. 2019; Kot et al. 2020b).

In the study by Preichardt et al. (2019), a culture medium based on sugarcane molasses (SCM) with added yeast extract (YE) and soybean meal (SM) was developed for the production of *Staphylococcus xylosus* AD1 biomass. A Box–Behnken optimization design was used for this purpose. Optimal culture conditions were obtained at concentrations of 10% SCM, 2% YE, and 4% SM, allowing for maximum live biomass growth. All media, except the one containing only SCM, showed better effectiveness in cultivating *S. xylosus* AD1 than the commercial Brain Heart Infusion medium. The medium with SCM, YE, and SM, according to Preichardt et al. (2019), is an excellent alternative for producing *S. xylosus* AD1 biomass.

In another study (Kot et al. 2020a), the possibility of using potato wastewater and glycerol fractions as media for the biosynthesis of lipids and carotenoids by the yeast *Rhodotorula gracilis* ATCC 10788 in a bioreactor was investigated. Cultivations were conducted at temperatures of 20 and 28 °C for 96 h. The highest lipid content (19 g/100 g biomass) was observed on the third and fourth days, with linoleic acid predominating (about 28%) after 96 h. The maximum carotenoid biosynthesis yield (6.24 mg/L) was achieved after 96 h, with β-carotene (about 47%) and torulene (about 51%) levels and negligible torularhodin (< 1%). The results indicate that *R. gracilis* biomass can be a good source of lipids and carotenoids, and the bioreactor cultivation method can reduce the environmental impact of industrial waste and starch-based biofuels.

The response surface methodology (RSM) is a timeless technique for optimizing biotechnological and microbiological processes, as evidenced by the presence of scientific articles spanning several decades to the present day. A good example is the study by Polak-Berecka et al. (2011), where RSM was used to investigate the effects of various medium components on the biomass production of *Lactobacillus rhamnosus* E/N. The best medium composition obtained by RSM contained (in g/L): glucose 15.44, sodium pyruvate 3.92, meat extract 8.0, potassium phosphate 1.88, sodium acetate 4.7, and ammonium citrate 1.88. With this medium composition, biomass production reached 23 g/L of dry cell mass after 18 h of cultivation in a bioreactor, while MRS medium yielded 21 g/L. The cost of 1 g of biomass on MRS was €0.44, while the new medium was 25% cheaper. The new, cheaper medium allows large-scale commercial cultivation of *L. rhamnosus*, which is significant for the commercial production of probiotic foods.

It has been shown that waste materials such as potato wastewater, glycerol fractions, sugarcane molasses, or corn steep liquor can effectively serve as inexpensive components of microbial media. By optimizing medium composition using RSM, it is possible not only to increase biomass yield or the production of desired metabolites but also to significantly reduce production costs. RSM is a promising approach to sustainable biotechnology, combining economic efficiency with minimizing environmental impact.

## Conclusions

The broad enzymatic capabilities of microbial transglutaminase have cemented its role as an essential tool in the industrial processing of foods and other materials. Its ability to improve texture, enhance nutritional value, and provide structural integrity to various products ensures that its applications will continue to expand. From food production to textile processing and biotechnology, MTG’s versatility ensures its growing role in industrial processes. As industries discover more uses for transglutaminase, its importance will grow across various sectors, especially as biotechnological advances continue. The statistical optimization of microbial transglutaminase (MTG) cultivation conditions can overcome the limitations of classical empirical methods. This study demonstrates an effective tool for optimizing MTG production by *Streptoverticillium cinnamoneum* KKP 1658. The research revealed interactions between independent variables and model responses. The most significant influence on transglutaminase production was observed for the cultivation time and the interaction between nitrogen dosage and cultivation time. The following optimal conditions for producing microbial transglutaminase at the laboratory scale using the *S. cinnamoneum* KKP 1658 strain were established: a 2% nitrogen source dosage combining aminobac with waste corn steep liquor, an initial cultivation pH range of 5.8–6.0 and a 48-h cultivation period. The experimental results underscore the importance of simultaneously investigating the effects of multiple factors on transglutaminase production owing to interactions between components, which can have a more significant impact than each factor individually. Further research aims to scale up the production of high-activity microbial transglutaminase (bioreactor production) by *S. cinnamoneum* KKP 1658.

## Data Availability

The data generated during and/or analyzed during the current study are available from the first author upon reasonable request.
